# Higher axillary lymph node metastasis burden in breast cancer patients with positive preoperative node biopsy: may not be appropriate to receive sentinel lymph node biopsy in the post-ACOSOG Z0011 trial era

**DOI:** 10.1186/s12957-019-1582-z

**Published:** 2019-02-20

**Authors:** Yue Liang, Xiaosong Chen, Yiwei Tong, Weiwei Zhan, Ying Zhu, Jiayi Wu, Ou Huang, Jianrong He, Li Zhu, Yafen Li, Weiguo Chen, Kunwei Shen

**Affiliations:** 1 Comprehensive Breast Health Center, Ruijin Hospital, Shanghai Jiaotong University School of Medicine, 197 Ruijin Er Road, Shanghai, 200025 China; 2 Department of Ultrasound Imaging, Ruijin Hospital, Shanghai Jiaotong University School of Medicine, Shanghai, China

**Keywords:** Breast cancer, Axillary lymph node metastasis, Fine-needle aspiration, Sentinel lymph node biopsy, Axillary lymph node dissection

## Abstract

**Background:**

Breast cancer patients with suspicious axillary lymph node (ALN) at ultrasound and positive fine-needle aspiration (FNA) results were required to receive ALN dissection (ALND), which was not certain in the post-ACOSOG Z0011 era. We aim to evaluate the ALN metastasis burden in these patients, thus to illustrate whether they can follow the ACOSOG Z0011 trial procedure.

**Methods:**

Clinically, T1–2 N0 breast cancer patients with positive preoperative ALN biopsy (FNA group) or 1–2 positive sentinel nodes (SLNB group) were retrospectively analyzed. ALN metastasis burden was compared between the two groups, which were further analyzed in certain subtypes. An association between clinicopathological factors and ≥ 3 ALN metastasis was also analyzed.

**Results:**

A total of 388 patients were included: 202 in the FNA group and 186 in the SLNB group. The FNA group had a significantly higher number of positive ALN (5.18 vs. 1.77, *P* <  0.001) and a larger proportion of patients with ≥ 3 ALN metastasis (58.42% vs. 11.83%, *P* <  0.001) than the SLNB group, which was not influenced by different tumor size stage and molecular subtypes. ALN metastasis identified by FNA was independently associated with a high rate of ≥ 3 ALN metastasis (OR = 6.98, 95% CI 1.95–25.02, *P* = 0.003).

**Conclusions:**

Patients with positive preoperative ALN biopsy had a higher ALN metastasis burden than patients with 1–2 positive SLNs, which was also the strongest factor associated with ≥ 3 ALN metastasis, indicating that these patients are not appropriate to receive SLNB in the post-ACOSOG Z0011 trial era.

**Electronic supplementary material:**

The online version of this article (10.1186/s12957-019-1582-z) contains supplementary material, which is available to authorized users.

## Background

Axillary lymph node (ALN) surgery is an important part of the surgical management of early breast cancer patients, which improves local disease control and guides further adjuvant systemic treatment [1, 2]. In practice, sentinel lymph node biopsy (SLNB) is firstly recommended for clinical ALN-negative patients. For patients with positive sentinel lymph node (SLN), axillary lymph node dissection (ALND) is the standard management for patients who do not receive breast-conserving surgery.

The methods of preoperative ALN evaluation include physical examination; imaging evaluation through ultrasound, mammogram, and MRI; fine-needle aspiration (FNA); and core needle biopsy (CNB) [3]. For patients with suspicious ALN at ultrasound, ultrasound-guided FNA is a convenient and accurate method for preoperative ALN evaluation [4, 5]. Patients with positive FNA results are recommended to receive ALND, which can avoid unnecessary SLNB [6, 7].

For patients with clinical T1–2 N0 disease, who have received breast-conserving surgery with 1–2 positive SLNs, the American College of Surgeons Oncology Group (ACOSOG) Z0011 trial has demonstrated that compared to SLNB alone, further ALND did not bring additional benefit in terms of loco-regional recurrence risk or overall survival [8–10]. The results of the Z0011 trial have thus led to the change of clinical ALN surgery management for these patients who meet the eligibility [11, 12]. In the post-ACOSOG Z0011 trial era, T1–2 N0 breast cancer patients with suspicious ALN at ultrasound and positive FNA results may also be eligible to receive SLNB and to omit ALND if they have no more than 2 positive SLNs, which challenge the role of preoperative ALN ultrasound evaluation.

In the current study, we aim to evaluate the ALN metastasis burden of T1–2 N0 patients with positive FNA results, which was further compared with those patients with 1–2 positive SLNs. Furthermore, clinical and pathological factors associated with ≥ 3 ALN metastasis were also analyzed, which may guide our further individualized ALN surgery management.

## Methods

### Patient population

**Fig. 1:**
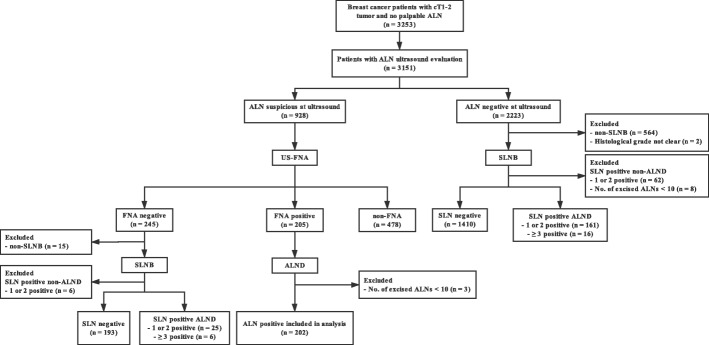
Flow chart of axillary lymph node preoperative evaluation and surgery. ALN axillary lymph node, US ultrasound, FNA fine-needle aspiration, SLN sentinel lymph node, SLNB sentinel lymph node biopsy, ALND axillary lymph node dissection

Breast cancer patients with clinical T1–2 tumor, no palpable ALN, who received surgery in the Comprehensive Breast Health Center, Ruijin Hospital, Shanghai Jiaotong University School of Medicine, between Jan. 2011 and May 2017 were retrospectively analyzed. All patients had preoperative ALN evaluation by physical examination and ALN ultrasound. Ultrasound-guided FNA was applied to patients with suspicious ALN at ultrasound. Patients with positive FNA results (FNA group) or 1–2 positive SLNs (SLNB group) were required to receive ALND. Other eligible criteria include invasive breast cancer, female gender, and not receiving preoperative therapy. Patients with clinically negative ALN but did not undergo SLNB, with positive SLN but did not undergo ALND, with < 10 ALNs excised during ALND, and with incomplete tumor histological information were excluded (Fig. 1). The independent Ethical Committee/Institutional Review Board of Ruijin Hospital, Shanghai Jiaotong University School of Medicine, reviewed and approved this study protocol, which was conducted in accordance with the Declaration of Helsinki.

### Preoperative ALN evaluation

A dedicated sonographer performed the ultrasound. Suspicious ALN at ultrasound was defined as nodes with round or irregular shape, diminished or absent hilum, or cortical thickness greater than 2 mm [8, 9]. The maximal cortical thickness was measured perpendicular to the long axis of the node on a cross-sectional plane, and for ALN without fatty hilum, the cortical thickness was measured as half the short axis of the node [13].

**Fig. 2:**
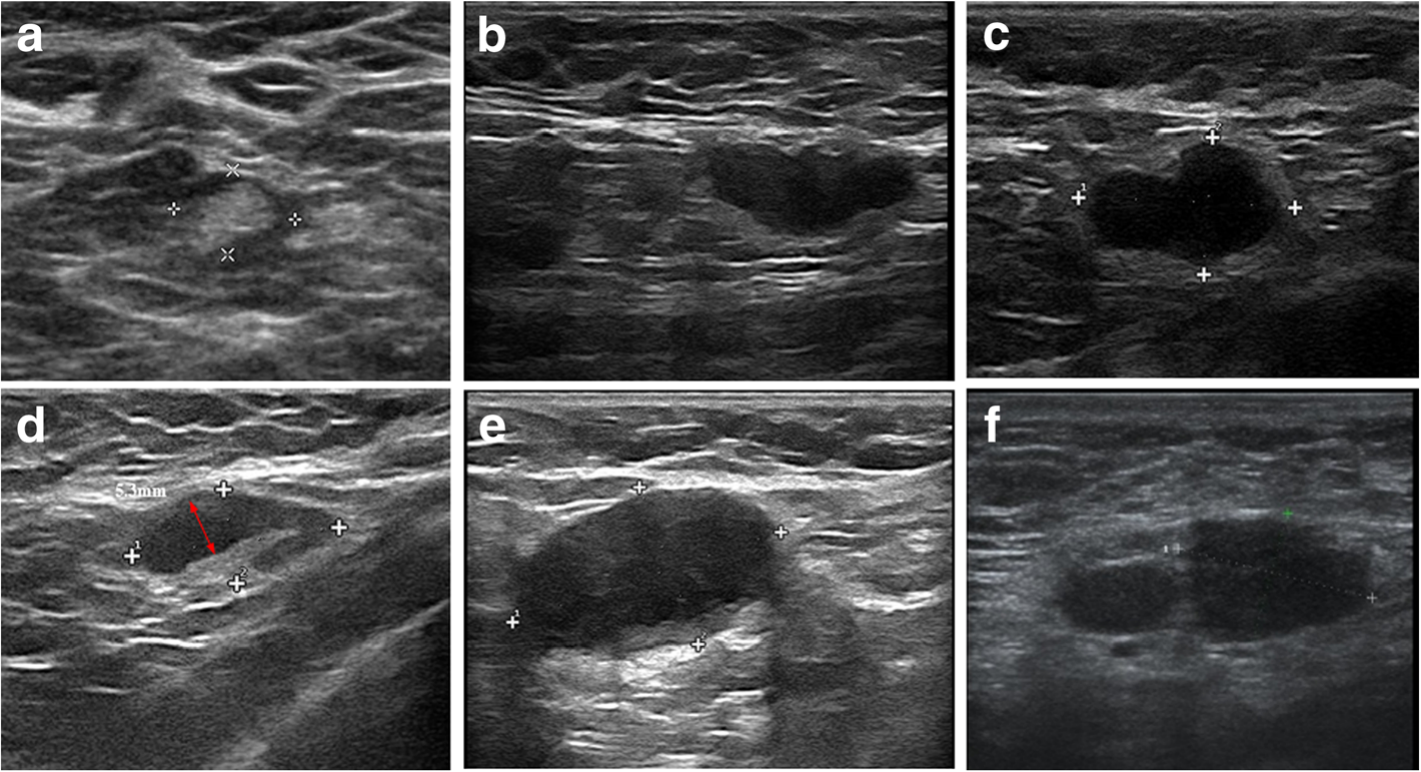
Diagrams of ALN at ultrasound. **a** Normal ALN. **b** Suspicious ALN with an irregular shape. **c** Suspicious ALN with absent hilum. **d** Suspicious ALN with a cortical thickness greater than 2 mm. **e** One suspicious ALN at ultrasound. **f** > 1 suspicious ALN at ultrasound. ALN axillary lymph node

For suspicious ALN at ultrasound, FNA was then performed to determine the cytopathologic diagnosis as we have previously described [7] (Fig. 2).

### Axillary surgery procedure

Patients with positive ALN identified by FNA were treated with ALND, while those with negative ALN at ultrasound or negative FNA results received SLNB. Figure 1 shows the ALN surgical procedure. Intraoperative SLN was evaluated by frozen pathologic testing. The seventh edition of the American Joint Committee on Cancer (AJCC) TNM staging system was used to classify ALN metastasis status [10]. Nodal metastasis was defined as the presence of macrometastasis (> 2.0 mm) or micrometastasis (> 0.2 but ≤ 2.0 mm). Lymph nodes with only isolated tumor cells (≤ 0.2 mm) were considered as negative.

### Data collection

Patients’ information was obtained from Shanghai Jiaotong University Breast Cancer Database (Copyright No. 015SR199280). Estrogen receptor (ER) status and progesterone receptor (PR) status were considered as positive if at least 1% of the tumor cells had nuclear staining. Hormonal receptor (HR) positivity was defined as ER/PR positive staining [14]. Tumors with immunohistochemical (IHC) HER2 2+ were further examined by fluorescent in situ hybridization (FISH). HER2 positivity was classified as IHC HER2 3+ or FISH+ [15]. Molecular subtypes were classified as luminal A (ER+/HER2−, PR ≥ 20%, Ki67 < 14%), luminal B-HER2 negative (ER+/HER2−, PR < 20%, and/or Ki67 ≥ 14%), luminal B-HER2 positive (HR+/HER2+), HER2 positive (HR−/HER2+), and triple negative (HR−/HER2−, TNBC) [16].

### Statistical analysis

Two sample *t* test, chi square, or two-tailed Fisher’s exact test were used to compare patient characteristics and ALN metastatic burden between the FNA group and the SLNB group and to compare patient characteristics between patients with 1–2 and ≥ 3 ALN metastasis. Binary logistic regression analysis was used to adjust patient characteristics for comparison of ALN metastatic burden between the two groups as well as factors associated with ≥ 3 ALN metastasis. The SPSS statistical software package, version 19.0 (SPSS, Chicago, IL, USA), was used for analysis, and a two-sided *P* value less than 0.05 was considered to be statistically significant.

## Results

### Patient characteristics and surgery

A total of 3253 patients with clinical T1–2 tumor and no palpable ALN received surgery between Jan. 2011 and May 2017, of which 3151 had preoperative ALN ultrasound evaluation. Ultrasound found suspicious ALN in 928 patients. FNA was conducted in 450 patients, among whom 202 had positive FNA results and received adequate ALND (FNA group). A total of 186 patients who had negative FNA results (*n* = 25) or negative ALN at ultrasound (*n* = 161) but 1–2 positive SLNs at surgery were included in the SLNB group. The ALN evaluation procedure is shown in Fig. 1. Mastectomy was performed in 71.51% (133/186) of patients with 1–2 positive SLNs and underwent ALND.

Clinicopathological characteristics of patients are listed in Table 1. Mean age was 54.38 years old. Compared with patients in the SLNB group, patients in the FNA group were more likely to have T2 tumors, > 1 suspicious ALNs at ultrasound, grade III tumors, and lymph-vascular invasion (LVI) positivity. Moreover, the FNA group had more tumors with ER negativity, PR negativity, HER2 positivity, high Ki67 expression, and HER2-positive subtype (*P* <  0.05). Multivariate analysis showed that patients in the FNA group were associated with > 1 suspicious ALN at ultrasound (OR = 51.30, 95% CI 26.60–98.91, *P* <  0.001), LVI positivity (OR = 4.87, 95% CI 2.12–11.18, *P* <  0.001), and high Ki67 expression (OR = 1.95, 95% CI 1.00–3.81, *P* = 0.049) compared with patients in the SLNB group (Additional file 1).

**Table 1:** Patient clinicopathological characteristics in the FNA and SLNB groups

Characteristics	All patients	FNA	SLNB	*P* value
	(*N* = 388) *N*	(*N* = 202) *N* (%)	(*N* = 186) *N* (%)	
Age [mean (range)] (year)	54.38 (31–84)	54.68 (31–84)	54.06 (31–83)	0.581
Tumor stage				0.021
T1	138	61 (30.20)	77 (41.40)	
T2	250	141 (69.80)	109 (58.60)	
Number of suspicious ALNs at US				< 0.001
≤ 1	207	37 (18.32)	170 (91.40)	
> 1	181	165 (81.68)	16 (8.60)	
Pathological type				0.391
Invasive ductal carcinoma	369	194 (96.04)	175 (94.09)	
Invasive lobular carcinoma	10	3 (1.49)	7 (3.76)	
Others	9	5 (2.48)	4 (2.15)	
Histological grade				0.001
I	15	4 (1.98)	11 (5.91)	
II	182	81 (40.10)	101 (54.30)	
III	191	117 (57.92)	74 (39.78)	
LVI				< 0.001
Negative	325	154 (76.24)	171 (91.94)	
Positive	63	48 (23.76)	15 (8.06)	
Multifocality				0.485
Unifocal	346	178 (88.12)	168 (90.32)	
Multifocal	42	24 (11.88)	18 (9.68)	
ER status				0.002
Negative	90	60 (29.70)	30 (16.13)	
Positive	298	142 (70.30)	156 (83.87)	
PR status				0.007
Negative	137	84 (41.58)	53 (28.49)	
Positive	251	118 (58.42)	133 (71.51)	
HER2 status				0.002
Negative	292	139 (68.81)	153 (82.26)	
Positive	96	63 (31.19)	33 (17.74)	
Ki67 (%, mean)				< 0.001
< 14%	114	41 (20.30)	73 (39.25)	
≥ 14%	274	161 (79.70)	113 (60.75)	
Molecular subtypes				0.003
Luminal A	78	31 (15.35)	47 (25.27)	
Luminal B-HER2 negative	179	88 (43.56)	91 (48.92)	
Luminal B-HER2 positive	42	23 (11.39)	19 (10.22)	
HER2 positive	54	40 (19.80)	14 (7.53)	
Triple negative	35	20 (9.90)	15 (8.06)	

*FNA* fine-needle aspiration, *SLNB* sentinel lymph node biopsy, *ALN* axillary lymph node, *US* ultrasound, *LVI* lymph-vascular invasion, *ER* estrogen receptor, *PR* progesterone receptor, *HER2* human epidermal growth factor receptor type 2

### ALN metastasis burden in the FNA and SLNB groups

All patients analyzed in this study were treated with ALND. The mean number of removed ALN was 19.96 in the FNA group and 19.03 in the SLNB group (*P* = 0.086). The mean number of positive ALN was 5.18 and 1.77 in the FNA and SLNB groups, respectively (*P* <  0.001). The proportion of patients with ≥ 3 ALN metastasis was 58.42% in the FNA group, which was much higher than those in the SLNB group (11.83%, *P* <  0.001) (Table 2). After adjusting clinicopathological factors, the FNA group was consistently associated with higher ALN metastasis burden compared with the SLNB group (*P* <  0.001).

**Table 2:** ALN metastasis burden between the FNA and SLNB groups

	Case no. (%)		*P* value
	FNA (*N* = 202)	SLNB (*N* = 186)	
Mean no. of ALN removed	19.96 (19.20–20.71)	19.03 (18.28–19.78)	0.086
Mean no. of positive ALN	5.18 (4.44–5.92)	1.77 (1.50–2.04)	< 0.001
No. of positive ALN			< 0.001
1+	33 (16.34)	119 (63.98)	
2+	51 (25.25)	45 (24.19)	
≥ 3+	118 (58.42)	22 (11.83)	

*ALN* axillary lymph node, *FNA* fine-needle aspiration, *SLNB* sentinel lymph node biopsy

### Comparison of ALN status in subgroups stratified by tumor stage

ALN status between the FNA and SLNB groups was further examined in different stage tumors. For patients with T1 tumor, the mean number of removed ALN was comparable between groups (19.38 vs. 18.30, *P* = 0.205). The mean number of positive ALN metastasis was significantly higher in the FNA group than in the SLNB group: 4.26 vs. 1.51, *P* <  0.001. Moreover, the proportion of patients with ≥ 3 ALN metastasis was much higher in the FNA group compared with the SLNB group: 52.46% vs. 6.49%, *P* <  0.001. Regarding patients with T2 tumor, there was also no significant difference in terms of the number of removed ALN between the two groups (*P* = 0.344). Patients in the FNA group had more ALN metastasis compared with those in the SLNB group (5.57 vs. 1.96, *P* <  0.001) and were more likely to have ≥ 3 ALN metastasis (60.99% vs. 15.60%, *P* <  0.001) (Table 3).

**Table 3:** ALN metastasis burden between the FNA and SLNB groups stratified by tumor stage

	T1			T2		
	FNA (*N* = 61)	SLNB (*N* = 77)	*P* value	FNA (*N* = 141)	SLNB (*N* = 109)	*P* value
No. of ALN removed [mean (95% CI)]	19.38 (18.07–20.69)	18.30 (17.21–19.39)	0.205	20.21 (19.28–21.13)	19.54 (18.52–20.57)	0.344
No. of positive ALN [mean (95% CI)]	4.26 (3.31–5.21)	1.51 (1.27–1.74)	< 0.001	5.57 (4.60–6.55)	1.96 (1.53–2.39)	< 0.001
No. of positive ALN			< 0.001			< 0.001
1+	13 (21.31)	52 (67.53)		20 (14.18)	67 (61.47)	
2+	16 (26.23)	20 (25.97)		35 (24.82)	25 (22.94)	
≥ 3+	32 (52.46)	5 (6.49)		86 (60.99)	17 (15.60)	

*ALN* axillary lymph node, *FNA* fine-needle aspiration, *SLNB* sentinel lymph node biopsy

### Comparison of ALN status in subgroups stratified by molecular subtype

Tumor stage and ALN metastasis burden were compared among molecular subtypes: HR+/HER2−, HER2+, and TNBC (Additional file 2). For each subtype, no difference of tumor stage was found between the FNA group and the SLNB group (*P* > 0.05). For luminal subtype and TNBC, there was no significant difference regarding removed ALN number between the FNA group and the SLNB group (*P* > 0.05), while HER2-positive patients in the FNA group had a higher number of removed ALN compared with those in the SLNB group (*P* = 0.007). In terms of the number of metastatic ALN, patients with HR+/HER2− or HER2+ tumors in the FNA group had more node metastasis compared with those in the SLNB group (*P* <  0.001). Moreover, there was no significant difference in the number of metastasis nodes between the two groups for patients with TNBC tumors (*P* = 0.338). However, irrespective of molecular subtypes, the FNA group had higher a proportion of patients with ≥ 3 ALN metastasis compared with the SLNB group (*P* <  0.001).

### Clinicopathological factors associated with ≥ 3 ALN metastasis

Univariate analysis found that tumor stage, grade, LVI status, multifocal status, HER2 status, and Ki67 expression level were significantly different between patients with 1–2 positive ALNs and ≥ 3 positive ALNs (*P* <  0.05). More patients with ≥ 3 ALN metastasis were in the FNA group than in the SLNB group (84.29% vs. 15.71%, *P* <  0.001). The number of suspicious ALN and cortical thickness of the suspicious ALN at ultrasound were related to the number of positive ALN (Additional file 3). In multivariate analysis, ALN metastasis identified by FNA was the strongest factor associated with ≥ 3 positive ALNs (OR = 6.98, 95% CI 1.95–25.02, *P* = 0.003; Table 4). Additionally, > 1 suspicious ALNs at ultrasound (OR = 5.38, 95% CI 2.31–12.56, *P* <  0.001), LVI positivity (OR = 4.78, 95% CI 2.04–11.24, *P* <  0.001), and multifocal tumors (OR = 3.93, 95% CI 1.33–11.63, *P* = 0.013) were independently associated with ≥ 3 ALN metastasis.

**Table 4:** Multivariate analysis of clinicopathological characteristics associated with ≥ 3 ALN metastasis

Characteristics	OR	95% CI	*P* value
ALN metastasis identified by			0.003
FNA	6.98	1.95–25.02	
SLNB	1.0		
Tumor stage			0.738
T1	1.0		
T2	1.12	0.57–2.21	
Number of suspicious ALNs at US			< 0.001
≤ 1	1.0		
> 1	5.38	2.31–12.56	
Thickness of the cortex at US (mm)			0.068
≤ 3.5	1.0		
> 3.5	1.81	0.96–3.41	
Histological grade			0.233
I	1.0		
II	0.31	0.03–3.36	0.335
III	0.50	0.05–5.40	0.570
LVI			< 0.001
Negative	1.0		
Positive	4.78	2.04–11.24	
Multifocality			0.013
Unifocal	1.0		
Multifocal	3.93	1.33–11.63	
HER2 status			0.932
Negative	1.0		
Positive	1.03	0.51–2.11	
Ki67 (%, mean)			0.567
< 14%	1.0		
≥ 14%	0.79	0.35–1.79	

*ALN* axillary lymph node, *OR* odds ratio, *CI* confidence interval, *FNA* fine-needle aspiration, *SLNB* sentinel lymph node biopsy, *US* ultrasound, *LVI* lymph-vascular invasion

## Discussion

Our current study found that T1–2 N0 breast cancer patients with suspicious ALN at ultrasound and positive FNA results (FNA group) had more ALN metastasis and a higher proportion of patients with ≥ 3 ALN metastasis compared with 1–2 SLN-positive patients (SLNB group), which was consistent in different tumor stage and molecular subtypes. A total of 58.42% patients in the FNA group had ≥ 3 ALN metastasis, indicating that these patients may not be appropriate for SLNB even in the post-ACOSOG Z0011 trial era. Meanwhile, ALN metastasis diagnosed by ultrasound-guided FNA was independently associated with ≥ 3 ALN metastasis (OR = 6.98), which was the leading risk factor among established clinicopathological factors, indicating that preoperative ALN ultrasound evaluation and ultrasound-guided FNA were still necessary for ALN management to spare two-step ALN surgery (ALND followed by positive SLNB) in the post-ACOSOG Z0011 trial era.

Preoperative ALN evaluation includes physical examination and axillary imaging. For patients with suspicious ALN at ultrasound, ultrasound-guided FNA or CNB was applied, which has been demonstrated to improve the accuracy of preoperative ALN evaluation. Patients with positive FNA results who will not receive neoadjuvant therapy are required to receive direct ALND. Since the ACOSOG Z0011 trial demonstrated ALND was not necessary for certain patients with 1–2 positive SLNs, then for patients with no palpable ALN but suspicious at ultrasound, SLNB is the first option and may spare further ALND if they were only found with 1–2 positive SLNs [8–10]. However, there were few studies on whether these patients with positive FNA results could be managed according to the ACOSOG Z0011 trial procedure. In the current study, we analyzed the SLNB group with 1–2 positive SLNs compared with the FNA group, and results showed that patients with positive FNA results had a higher node metastasis burden than those in the SLNB group. Previous study found that 89% of the patients with suspicious ALN at ultrasound and positive FNA results were not eligible for the ACOSOG Z0011 trial, and 48% of these patients had ≥ 3 ALN metastasis [17]. Farrell et al. demonstrated that the number of ALN metastasis was much higher in patients with suspicious ALN at ultrasound and positive FNA results than those diagnosed by SLNB (5.2 vs. 2.2) [18]. Boland et al. found that 61% patients with suspicious ALN at ultrasound and positive FNA results had more than 2 ALN metastasis [19]. Our findings were consistent with the above studies in terms of the mean number of positive ALN and proportion of patients with ≥ 3 ALN metastasis, which was much higher in the FNA group than in the SLNB group, indicating that patients in the FNA group without detailed selection may not be suitable to perform SLNB according to the ACOSOG Z0011 trial result.

When comparing the clinicopathological factors between the FNA and SLNB groups, we found that patients with positive FNA results were associated with > 1 suspicious ALNs at ultrasound, LVI, and higher Ki67 expression. Such association was consistent with the previous reports which can predict the ALN metastasis burden. Hieken et al. reported that the proportion of patients with stage N2 disease was significantly higher in patients with > 1 suspicious than 1 suspicious ALN at ultrasound (31% vs. 14%, *P* <  0.001) [20]. Pilewskie et al. demonstrated a higher proportion of patients with ≥ 3 ALN metastasis in patients with > 1 suspicious than 1 suspicious ALN at ultrasound (68% vs. 43%, *P* = 0.003) [21]. LVI positivity [22] and high Ki67 expression were also reported to be independently associated with > 3 ALN metastasis [23, 24].

Since tumor stage and molecular subtypes were reported to be associated with ALN metastasis [25, 26], we further analyzed the ALN metastasis burden between the FNA and SLNB groups in the above subgroups. Our study found that ALN metastasis burden was significantly higher in the FNA group than in the SLNB group irrespective of molecular subgroups, indicating that routine clinicopathological factors may not be enough to select FNA-positive patients to receive ALN surgery according to the ACOSOG Z0011 trial procedure.

There were several clinicopathological factors associated with ≥ 3 ALN metastasis, which could help us choose proper patients to receive certain ALN surgery. In our study, we found that ALN metastasis identified by FNA, > 1 suspicious ALNs at ultrasound, LVI positivity, and multifocality were independently associated with ≥ 3 ALN metastasis, which was consistent with previous studies [27]. To note, node metastasis diagnosed by FNA was the most important factor to predict ≥ 3 ALN metastasis (OR = 6.98). Caudle et al. reported that ALN metastasis identified by FNA was the independent predictive factor of ≥ 3 ALN metastasis in clinical T1–2 patients. They also showed that > 1 suspicious ALNs at ultrasound, LVI positivity, and multifocality were also associated with ≥ 3 ALN metastasis [27]. Although results of the ACOSOG Z0011 trial have changed our ALN surgery procedure, FNA is still valuable in selecting patients more likely with ≥ 3 ALN metastasis and one-step ALND is a good option for these patients in the FNA group.

Since FNA-positive results were associated with higher ALN metastasis burden, it was useful in the management of ALN in the era of post-ACOSOG Z0011 trial. Moreover, patients with positive ALN FNA results were more likely to receive neoadjuvant chemotherapy, especially for TNBC or HER2+ breast cancer. Furthermore, after neoadjuvant chemotherapy, SLNB should be performed with caution for these ALN FNA+ breast cancer patients. In our cohort, 67 (24.63%) ALN FNA+ breast cancer patients were recommended to receive neoadjuvant chemotherapy: 29 (43.28%) patients with HER2+ breast cancer and 7 (10.45%) patients with TNBC.

Our study had several limitations. Firstly, patients with positive FNA results may be treated with neoadjuvant therapy, especially for those with relatively larger tumor or HER2+/TNBC subtypes. And we excluded 68 patients with 1–2 positive SLNs who did not receive ALND according to the ACOSOG Z0011 trial model, which will cause selection bias in the FNA group. Secondly, the 10-year follow-up of the AMAROS trial demonstrated equivalent results of long-term survival and loco-regional recurrence between ALND and regional irradiation in patients with T1–2 tumor and no palpable lymphadenopathy. One-step ALND may be not appropriate for all cT1–2 non-palpable ALN breast cancer patients but with FNA+ results, which needs to be discussed and can be treated according to the AMAROS trial procedure, which may reduce the complication of ALND. In addition, patient number was relatively insufficient in certain subgroups in terms of tumor size stage and molecular subtype, and a larger sample size is necessary to validate our findings. Last but not the least, further analysis in regard to the survival outcome difference between the two groups is warranted, so as to help choose the proper ALN surgery procedure for these patients.

## Conclusions

Our study demonstrated that T1–2 N0 breast cancer patients in the FNA group had higher node metastasis burden compared with those in the SLNB group. FNA-positive patients had more positive ALNs and a higher rate of ≥ 3 ALN metastasis, regardless of tumor size stage and molecular subtypes. ALN metastasis identified by FNA was the strongest predictive factor of ≥ 3 ALN metastasis, indicating that preoperative ultrasound ALN evaluation was still necessary in clinical practice. FNA-positive patients, if not selected, were not appropriate to firstly receive SLNB according to the ACOSOG Z0011 trial procedure, which warrants further clinical study.

## Additional files

Additional file 1: Multivariate analysis of clinicopathological characteristics associated with the FNA group compared with the SLNB group. (DOCX 15 kb)

Additional file 2: ALN metastasis burden between the FNA and SLNB groups stratified by molecular subtypes. (DOCX 14 kb)

Additional file 3: Clinicopathological characteristics between patients with 1–2 and ≥ 3 ALN metastasis. (DOCX 16 kb)

## Abbreviations

ACOSOG: American College of Surgeons Oncology Group
AJCC: American Joint Committee on Cancer
ALN: Axillary lymph node
ALND: Axillary lymph node dissection
CNB: Core needle biopsy
ER: Estrogen receptor
FISH: Fluorescent in situ hybridization
FNA: Fine-needle aspiration
HR: Hormonal receptor
IHC: Immunohistochemical
LVI: Lymph-vascular invasion
PR: Progesterone receptor
SLN: Sentinel lymph node
SLNB: Sentinel lymph node biopsy
