# Supplementary material for: Higher axillary lymph node metastasis burden in breast cancer patients with positive preoperative node biopsy: may not be appropriate to receive sentinel lymph node biopsy in the post-ACOSOG Z0011 trial era
Source: World J Surg Oncol. 2019 Feb 20;17:37. doi: 10.1186/s12957-019-1582-z (PMC6383227; doi:10.1186/s12957-019-1582-z)
Supplement: Supplementary file 1 — Multivariate analysis of clinicopathological characteristics associated with the FNA group compared with the SLNB group. (DOCX 15 kb) [file 12957_2019_1582_MOESM1_ESM.docx]

**Additional file 1. Multivariate analysis of** **clinicopathologic characteristics associated with the FNA group compared with the SLNB group**

| **Characteristics** | **Odds ratio** | **95%** **confidence interval** | **P value** |
| --- | --- | --- | --- |
| **Tumor stage** |  |  | 0.855 |
| T1 | 1.0 |  |  |
| T2 | 0.94 | 0.49-1.80 |  |
| **Number of suspicious ALNs at ultrasound** |  |  | < 0.001 |
| ≤ 1 | 1.0 |  |  |
| > 1 | 51.30 | 26.60-98.91 |  |
| **Histological grade** |  |  | 0.488 |
| I | 1.0 |  |  |
| II | 1.59 | 0.28-9.06 | 0.60 |
| III | 2.27 | 0.37-13.84 | 0.374 |
| **LVI** |  |  | < 0.001 |
| Negative | 1.0 |  |  |
| Positive | 4.87 | 2.12-11.18 |  |
| **ER status** |  |  | 0.956 |
| Negative | 0.97 | 0.34-2.79 |  |
| Positive | 1.0 |  |  |
| **Progesterone receptor status** |  |  | 0.298 |
| Negative | 1.43 | 0.73-2.78 |  |
| Positive | 1.0 |  |  |
| **HER2 status** |  |  | 0.467 |
| Negative | 1.0 |  |  |
| Positive | 0.74 | 0.33-1.66 |  |
| **Ki67 (%, mean)** |  |  | 0.049 |
| < 14% | 1.0 |  |  |
| ≥ 14% | 1.95 | 1.00-3.81 |  |
| **Molecular subtypes** |  |  | 0.921 |
| Luminal A | 1.0 |  |  |
| Luminal B-HER2 negative | 1.06 | 0.30-3.75 | 0.933 |
| Luminal B-HER2 positive | 0.80 | 0.18-3.69 | 0.777 |
| HER2 positive | 0.69 | 0.12-4.17 | 0.687 |
| Triple negative | 1.21 | 0.20-7.14 | 0.837 |

*ALN* axillary lymph node, *FNA* fine-needle aspiration, *SLNB* sentinel lymph node biopsy, *LVI* lymphvascular invasion, *ER* estrogen receptor, *HER2* human epidermal growth factor receptor type 2
