# Supplementary material for: Higher axillary lymph node metastasis burden in breast cancer patients with positive preoperative node biopsy: may not be appropriate to receive sentinel lymph node biopsy in the post-ACOSOG Z0011 trial era
Source: World J Surg Oncol. 2019 Feb 20;17:37. doi: 10.1186/s12957-019-1582-z (PMC6383227; doi:10.1186/s12957-019-1582-z)
Supplement: Supplementary file 2 — ALN metastasis burden between the FNA and SLNB groups stratified by molecular subtypes. (DOCX 14 kb) [file 12957_2019_1582_MOESM2_ESM.docx]

**Additional file 2. ALN metastasis burden between the FNA and SLNB groups stratified by molecular subtypes**

|  | **HR+/HER2-** | | **P value** | **HER2+** | | **P value** | **HR-/HER2-** | | **P value** |
| --- | --- | --- | --- | --- | --- | --- | --- | --- | --- |
|  | **FNA (N = 119)** | **SLNB (N = 138)** |  | **FNA (N = 63)** | **SLNB (N = 33)** |  | **FNA (N = 20)** | **SLNB (N = 15)** |  |
| **Tumor stage** |  |  | 0.122 |  |  | 0.774 |  |  | 0.094 |
| T1 | 43 (36.13) | 63 (45.65) |  | 15 (23.81) | 7 (21.21) |  | 3 (15.00) | 7 (46.67) |  |
| T2 | 76 (63.87) | 75 (54.35) |  | 48 (76.19) | 26 (78.79) |  | 17 (85.00) | 8 (53.33) |  |
| **No. of ALN** **removed [mean (95% confidence interval)]** | 19.55 (18.71-20.38) | 19.19 (18.28-20.10) | 0.572 | 21.11 (19.44-22.78) | 18.06 (16.57-19.55) | 0.007 | 18.75 (16.21-21.29) | 19.67 (16.90-22.43) | 0.612 |
| **No. of positive ALN [mean (95% confidence interval)]** | 4.91 (4.01-5.81) | 1.77 (1.47-2.07) | < 0.001 | 6.13 (4.58-7.68) | 1.52 (1.26-1.77) | < 0.001 | 3.80 (1.67-5.93) | 2.40 (0.34-4.46) | 0.338 |
| **No. of positive ALN** |  |  | < 0.001 |  |  | < 0.001 |  |  | 0.005 |
| 1+ | 21 (17.65) | 88 (63.77) |  | 8 (12.70) | 20 (60.61) |  | 4 (20.00) | 11 (73.33) |  |
| 2+ | 30 (25.21) | 34 (24.64) |  | 15 (23.81) | 9 (27.27) |  | 6 (30.00) | 2 (13.33) |  |
| ≥ 3+ | 68 (57.14) | 16 (11.59) |  | 40 (63.49) | 4 (12.12) |  | 10 (50.00) | 2 (13.33) |  |

*ALN* axillary lymph node, *FNA* fine-needle aspiration, *SLNB* sentinel lymph node biopsy, *HER2* human epidermal growth factor receptor type 2
