# Supplementary material for: Higher axillary lymph node metastasis burden in breast cancer patients with positive preoperative node biopsy: may not be appropriate to receive sentinel lymph node biopsy in the post-ACOSOG Z0011 trial era
Source: World J Surg Oncol. 2019 Feb 20;17:37. doi: 10.1186/s12957-019-1582-z (PMC6383227; doi:10.1186/s12957-019-1582-z)
Supplement: Supplementary file 3 — Clinicopathological characteristics between patients with 1–2 and ≥ 3 ALN metastasis. (DOCX 16 kb) [file 12957_2019_1582_MOESM3_ESM.docx]

**Additional file 3. Clinicopathologic characteristics between patients with 1-2 and ≥ 3 ALN metastasis**

| **Characteristics** | **Case No. (%)** | |  |
| --- | --- | --- | --- |
|  | **ALN 1-2+**  **(N = 248)** | **ALN ≥ 3+**  **(N = 140)** | **P value** |
| **ALN metastasis identified by** |  |  | < 0.001 |
| FNA | 84 (33.87) | 118 (84.29) |  |
| SLNB | 164 (66.13) | 22 (15.71) |  |
| **Age [mean (range)] (year)** | 54.65 (31-84) | 53.91 (31-78) | 0.508 |
| **Tumor stage** |  |  | 0.005 |
| T1 | 101 (40.73) | 37 (26.43) |  |
| T2 | 147 (59.27) | 103 (73.57) |  |
| **Number of suspicious ALNs at ultrasound** |  |  | < 0.001 |
| ≤ 1 | 175 (70.56) | 32 (22.86) |  |
| > 1 | 73 (29.44) | 108 (77.14) |  |
| **Fatty hilum at axillary ultrasound*** |  |  | 0.463 |
| absent | 38 (36.54) | 50 (41.32) |  |
| present | 66 (63.46) | 71 (58.68) |  |
| **Thickness of the cortex at ultrasound (mm)*** |  |  | 0.037 |
| ≤ 3.5 | 43 (41.35) | 34 (28.10) |  |
| > 3.5 | 61 (58.65) | 87 (71.90) |  |
| **Pathological type** |  |  | 0.522 |
| Invasive ductal carcinoma | 234 (94.35) | 135 (96.43) |  |
| Invasive lobular carcinoma | 8 (3.23) | 2 (1.43) |  |
| Others | 6 (2.42) | 3 (2.14) |  |
| **Histological grade** |  |  | 0.002 |
| I | 12 (4.84) | 3 (2.14) |  |
| II | 130 (52.42) | 52 (37.14) |  |
| III | 106 (42.74) | 85 (60.71) |  |
| **LVI** |  |  | < 0.001 |
| Negative | 225 (90.73) | 100 (71.43) |  |
| Positive | 23 (9.27) | 40 (28.57) |  |
| **Multifocality** |  |  | 0.003 |
| Unifocal | 230 (92.74) | 116 (82.86) |  |
| Multifocal | 18 (7.26) | 24 (17.14) |  |
| **ER status** |  |  | 0.102 |
| Negative | 51 (20.56) | 39 (27.86) |  |
| Positive | 197 (79.44) | 101 (72.14) |  |
| **Progesterone receptor status** |  |  | 0.058 |
| Negative | 79 (31.85) | 58 (41.43) |  |
| Positive | 169 (68.15) | 82 (58.57) |  |
| **HER2 status** |  |  | 0.022 |
| Negative | 196 (79.03) | 96 (68.57) |  |
| Positive | 52 (20.97) | 44 (31.43) |  |
| **Ki67 (%, mean)** |  |  | 0.019 |
| < 14% | 83 (33.47) | 31 (22.14) |  |
| ≥ 14% | 165 (66.53) | 109 (77.86) |  |
| **Molecular subtypes** |  |  | 0.098 |
| Luminal A | 57 (22.98) | 21 (15.00) |  |
| Luminal B-HER2 negative | 116 (46.77) | 63 (45.00) |  |
| Luminal B-HER2 positive | 25 (10.08) | 17 (12.14) |  |
| HER2 positive | 27 (10.89) | 27 (19.29) |  |
| Triple negative | 23 (9.27) | 12 (8.57) |  |

*ALN* axillary lymph node, *FNA* fine-needle aspiration, *SLNB* sentinel lymph node biopsy, *LVI* lymph vascular invasion, *ER* estrogen receptor, *HER2* human epidermal growth factor receptor type 2

*225 patients with suspicious ALN at ultrasound were evaluated for this characteristic
